# 3,3′-Diindolylmethane enhances apoptosis in docetaxel-treated breast cancer cells by generation of reactive oxygen species

**DOI:** 10.1080/13880209.2018.1495747

**Published:** 2018-10-10

**Authors:** Susan Lanza-Jacoby, Guanjun Cheng

**Affiliations:** Department of Surgery, Thomas Jefferson University, Philadelphia, PA, USA

**Keywords:** Chemoresistance, apoptosis, oxidative stress, plant compounds, chemotherapy

## Abstract

**Context:** A major problem in the treatment of cancer is the development of toxic side effects and resistance to chemotherapy. The use of plant compounds to overcome resistance and prevent toxicity is a potential strategy for treatment.

**Objective:** We evaluated whether 3,3′-diindolylmethane (DIM) enhanced the sensitivity of breast cancer cells to docetaxel (DOC).

**Materials and methods:** MDA-MB231 and Sk-BR-3 cells were treated with and without 25 or 50 µM of DIM and 1 nM of DOC for 48 and 72 h, respectively. MTT assay was used to measure cell survival. Apoptosis and intracellular reactive oxygen species (ROS) were determined by flow cytometry. The expression of proteins regulating ROS production and apoptosis was evaluated by immunoblotting technique.

**Results:** Combining 25 µM of DIM with 1 nM DOC decreased cell survival by 42% in MDA-MB231 cells and 59% in Sk-BR-3 cells compared to control, DIM, or DOC (*p* ≤ 0.05). The combination treatment increased apoptosis over 20% (*p* ≤ 0.01) in both cell lines, which was associated with decreased Bcl-2, increased Bax, cleaved PARP and activated JNK (*p* ≤ 0.01). ROS production increased by 46.5% in the MDA-MB231 and 29.3% in Sk-BR-3 cells with the combination compared to DIM or DOC alone. Pretreating cells with *N*-acetyl-cysteine or Tiron abrogated the anti-survival effect of the combination. The increase in ROS was associated with a 54% decrease in MnSOD and 47% increase in NOX2 protein compared to the other groups.

**Conclusions:** Our findings indicated that DIM enhances the sensitivity of breast cancer cells to DOC treatment by increasing ROS, which led to decreased cell survival and apoptosis.

## Introduction

Numerous studies have demonstrated the protective effects of dietary components in vegetables for cancer prevention. Although supplement use has been increased in cancer patients in recent years, there is very little information regarding the effect of these compounds during chemotherapy. Indole-3-carbinol (I3C) is one such naturally occurring active component of cruciferous vegetables. The crushing or mechanical chewing of the vegetable releases plant enzymes that break down the glucosinolates found in all cruciferous vegetables to I3C, which, in turn, is further broken down in the acidic pH of the stomach to several condensed products including 3,3′-diindolylmethane (DIM). Plasma from humans fed with I3C supplements contains DIM and not I3C, which suggests that DIM is the active component that reaches the cells (Bell et al. 2000). DIM supplements are commercially available and inexpensive. I3C and DIM have been shown to protect against cancers of prostate (Chinni et al. 2001), cervix (Chen et al. 2001), breast (Hong et al. 2002), colon (Kim et al. 2009), pancreas (Azmi et al. 2008) and lung (Kassie et al. 2007). Studies have demonstrated that DIM inhibits proliferation, cell cycle and induces apoptosis in estrogen receptor negative (ER–) and ER positive (ER+) breast cancer cells (Hong et al. 2002). DIM was found to inhibit tumor growth by regulating nuclear factor-κΒ (NF-κΒ) (Rahman and Sarkar 2005), FOXM1 (Ahmad et al. 2011) cdk6 and p21 (Firestone and Bjeldanes 2003). Previous studies have also shown that DIM induced stress-activated pathways including activating JNK kinase, which are important in regulating cell growth and apoptosis (Xue et al. 2005; Gong et al. 2006). These studies have identified a role for oxidative stress by DIM in mediating G2 cell cycle arrest (Chen et al. 1998), decreasing ATP synthase leading to the induction of p21 (Gong et al. 2006) and inhibiting FOF-ATP synthase resulting in depletion of ATP and induction of ROS (Roy et al. 2008).

Docetaxel (DOC) is the most widely used taxane for the treatment of several cancers including breast, lung, squamous cell carcinoma of the head and neck, ovary and the prostate. Considering that DOC is more toxic than paclitaxel and is very often administered with other chemotherapeutic drugs, the problem of toxicity increases with many patients experiencing unpleasant side effects. There are reports which indicate that the plant compounds such as curcumin (Bayet-Robert et al. 2010), green tea (Luo et al. 2010) and resveratrol (Al-Abd et al. 2011) increase the sensitivity of breast cancers to DOC with the potential for reducing toxicity. In an earlier study, we showed that DIM enhanced apoptosis in paclitaxel-treated 435eB1 breast cancer cells (McGuire et al. 2006). Our findings were confirmed by other reports (Rahman et al. 2007; Ahmad et al. 2011). However, the mechanism by which DIM enhances the anticancer effect of taxanes in breast cancer is not well understood. Previous studies have demonstrated that the treatment of cancer cells with DIM induced ROS production (Gong et al. 2006; Kandala and Srivastava 2010; Wang et al. 2016). Considering these observations, we investigated whether ROS is a mechanism by which DIM sensitizes breast cancer cells to the chemotherapeutic agent, DOC.

## Materials and methods

### Cell culture and reagents

The human breast cancer cell lines MDA-MB231 and SK-BR-3 were obtained from American Type Culture Collection (ATCC), Manassas, VA. MDA-MB231 cells are a triple negative, highly aggressive cell line and SK-BR-3 cells are ER- and overexpress HER-2. Cells were cultured in Dulbecco’s Modified Eagle’s Medium (DMEM) containing 1X penicillin/streptomycin and supplemented with 10% fetal bovine serum (Atlantic Biologicals, Miami, FL) in a humidified atmosphere with 5% CO_2_ at 37 °C. To remove the adherent cells from the flasks for passaging or counting, cells were washed with Hanks’ balanced salt solution without calcium or magnesium, then the cells were incubated with a small volume of 0.25% trypsin-EDTA (Sigma Chemical Co., St. Louis, MO) for 5–10 min and washed with culture medium by centrifugation. We obtained BioResponse DIM from Michael Zelsnick, Bio Response, Boulder, CO and DOC was obtained from Aventis Pharmaceuticals, Bridgewater, NJ.

For immunoblot analysis, the following antibodies were used: Bcl-2, Bax, poly-ADP-ribose polymerases (PARP), cleaved PARP, JNK, phosphorylated JNK (Cell Signaling Technology, Beverly, MA), MnSOD (Santa Cruz, Santa Cruz, CA), NOX4 and NOX2 (Novus Biologicals, Littleton, CO) and GAPDH (Millipore Corp., Billerica, MA).

### Cell viability

The viability of cells was determined by the mitochondrial reductase activity as an indicator of viable cells; assays were conducted with 3-(4,5-dimethylthiazol-2-yl)-2,5-diphenyltetrazolium bromide (MTT) reduction assay. Cells were seeded on 96-well plates at a density of 1 × 10^4^ cells/well and allowed to adhere for 24 h. Then, the cells were incubated with DIM, DOC, or their combinations. After the required incubation time, the wells were vacuumed and treated with 0.25 mg/mL MTT dissolved in DMSO for 3 h at 37 °C. A microplate reader was used to measure the absorbance at a wavelength of 570 nm.

#### Immunoblotting

Cells were washed with cold PBS and harvested by scraping into 0.2 mL of RIPA buffer containing 50 mM Tris-HCl (pH 7.4), 150 mM NaCl, 1% NP40, 1 mM EDTA, 0.25% sodium deoxycholate, 1 mM NaF, 10 µM Na_3_VO_4_, 1 mM phenylmethysulfonyl fluoride and protease inhibitor cocktail (Sigma Chemical Co, St Louis, MO, catalog # P7626 and P8340) according to manufacturer’s instructions. Protein concentration of cell lysates was determined by using a Bio-Rad protein assay (Bio-Rad, Hercules, CA). Proteins were denatured by heat at 95 °C and separated by 3–8% NuPAGE gel or 4–12% NuPAGE and transferred to a nitrocellulose membrane and nonspecific binding was blocked with 5% dry milk in TBST (40 mM Tris-Cl, pH 7.6, 150 mM, NaCl, 0.2% Tween-20) for 1 h at room temperature. With constant shaking, membranes were incubated with primary antibodies overnight at 4 °C. After washing with TBST three times, the membranes were incubated with secondary antibodies at room temperature for 1 h with constant shaking. The expression of the targeted proteins was detected using an ECL kit (Amersham Biosciences, Marlborough, MA) following manufacturer’s instructions and visualized by autoradiography with Hyperfilm.

#### Annexin V-FITC staining

Apoptotic cells were quantified using the annexin V plus propidium Iodide Apoptosis detection kit (R & D Systems, Minneapolis, MN) according to manufacturer’s instructions. Approximately 5 × 10^5^ cells were resuspended in 100 µL of the manufacturer-supplied 1x binding buffer and mixed with 1 µL of annexin V-FITC and 10 µL of propidium iodide. After 15 min of incubation in the dark at room temperature, 400 µL of 1x binding buffer was added and the cells were analyzed using a BD FACS flow cytometer. Annexin V detects cellular apoptosis, while propidium iodide detects necrotic or late apoptotic cells.

### Measurement of ROS production

Prooxidant levels (H_2_O_2_) were measured by labeling cells for 15 min at 37 °C using oxidation sensitive [C-400; 5- (and-6)-carboxy-2′,7′-dichlorodihydrofluorescein diacetate (DCFDA) Molecular Probes, Eugene, OR] and evaluating the conversion to [C-369; 5-(and-6)-carboxy-2′,7′-dichlorofluorescein diacetate] (DCF), a fluorescent dye. Cells were seeded into six-well culture plates at a density of 5 × 10^5^/mL and treated for 24 or 48 h with the indicated concentrations of DIM with and without DOC. For analysis, cells were trypsinized, washed in warm PBS, incubated with 10 µM DCFDA for 30 min and analyzed by BD FACS flow cytometer. In some experiments the antioxidant, N-acetyl cysteine (NAC) or Tiron (superoxide scavenger) were added to the cells that were pretreated for 1 h with either 5 mM NAC or 1 mM Tiron and incubated for 24 or 48 h.

### Statistical analysis

Results are expressed as mean ± SE of 3–4 different experiments. Statistical analysis was performed using one or two-way ANOVA followed by the Bonferroni *post-hoc* test using SAS 9.3 software analysis (SAS Institute, Cary, NC, USA).

## Results

### DIM in combination with DOC decreased the viability of human breast cancer cells

**Figure 1.**
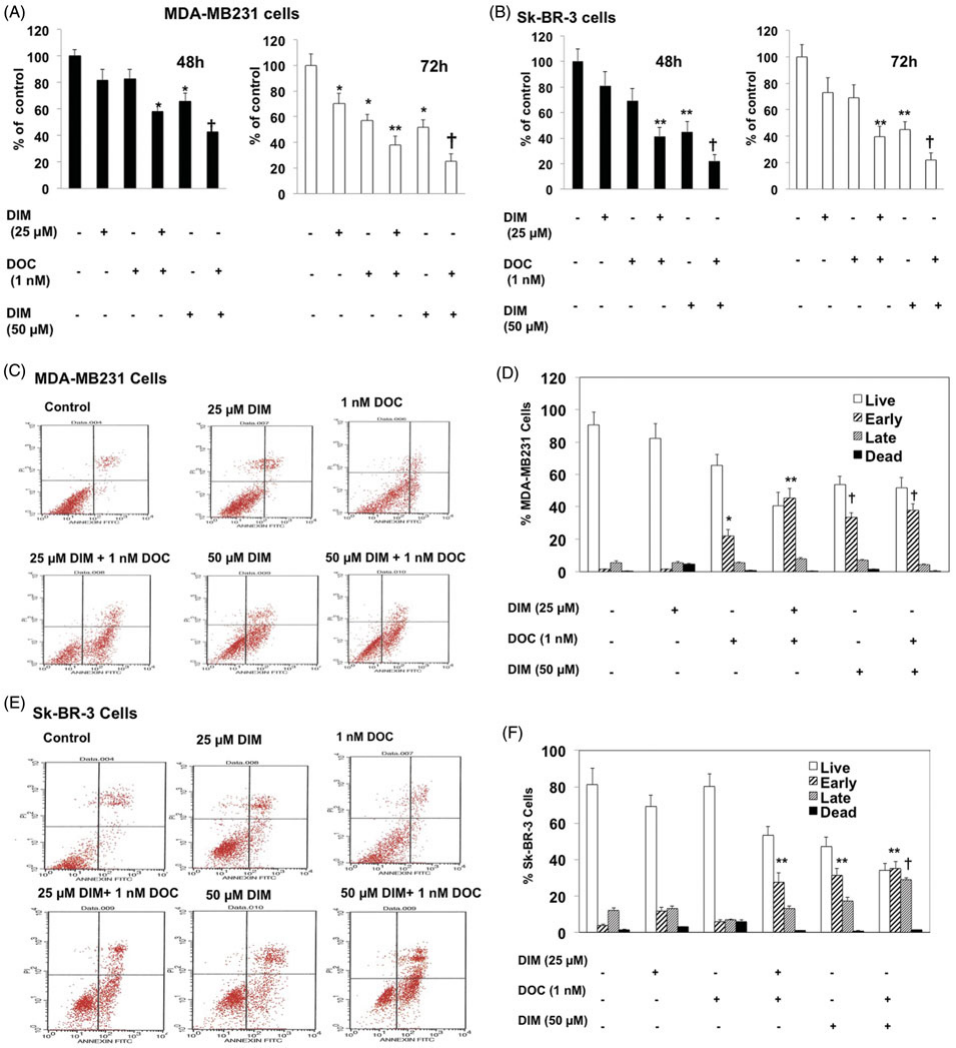
DIM decreased the viability and enhances apoptosis of breast cancer cells treated with docetaxel (DOC). (A) MDA-MB231 and (B) SkBR3 cells were treated for 48 or 72 h with the indicated concentrations of DIM with or without 1 nM DOC. Cell viability was determined by the MTT assay. (C, D) MDA-MB231 and (E, F) SkBR3 cells were treated for 48 h, harvested, and subject to annexin V-FITC plus propidium iodide staining. Apoptosis was analyzed by flow cytometry and normalized to untreated controls. Data are calculated as percent of control and expressed as the mean percentage of control ± SE of 3 independent experiments. *p* Values were determined using ANOVA. Bars with different symbols are significantly different (*, *p* < 0.05 vs. control, **, *p* < 0.01 vs. control, 25 µM DIM alone, DOC alone; **†**, *p* < 0.001 vs. control, 25 µM DIM alone, DOC alone, and 25 µM DIM plus DOC).

MDA-MB231 and Sk-BR-3 cells were incubated with DIM (25 or 50 µM) alone or in combination with 1 nM DOC for 48 and 72 h. The concentrations of DIM and DOC were selected based on previous studies demonstrating the cytotoxicity in these cells and other breast cancer cells (Rahman et al. 2007; Ahmad et al. 2011). After 48 h of treatment, cell survival did not decrease significantly with 25 µM DIM or 1 nM DOC treatment alone, whereas increasing DIM concentration to 50 µM decreased the survival (Figure 1(A,B)). After 72 h, single treatments of DIM or DOC alone decreased survival in MDA-MB231 but not in Sk-BR-3 cells. However, when 25 µM of DIM was combined with 1 nM DOC and treated for 48 h, cell survival decreased by 42% (*p* ≤ 0.05) in MDA-MB231 cells and 59% in Sk-BR-3 cells (*p* ≤ 0.01) compared to control or either agent alone. The results of the combination appear to induce synergistic cytotoxicity. The anti-survival effect of the treatments increased with a longer incubation time from 48 to 72 h in MDA-MB231 cells but not in Sk-BR-3 cells, which suggests resistance to the treatments. Sk-BR-3 cells overexpress HER2 gene, which has been shown to confer resistance to DOC with respect to cell viability and apoptosis (Yu et al. 1996; Carpenter and Lo 2013). HER2 activates PI3K/Akt/ERK signaling, resulting in proliferation and inhibition of apoptosis. Combining 50 µM DIM with 1 nM DOC produced an even greater growth-inhibition (*p* ≤ 0.001) than the combination of the lower concentration of DIM (25 µM) with DOC in both cell lines. DIM enhanced the apoptotic effects of DOC.

**Figure 2.**
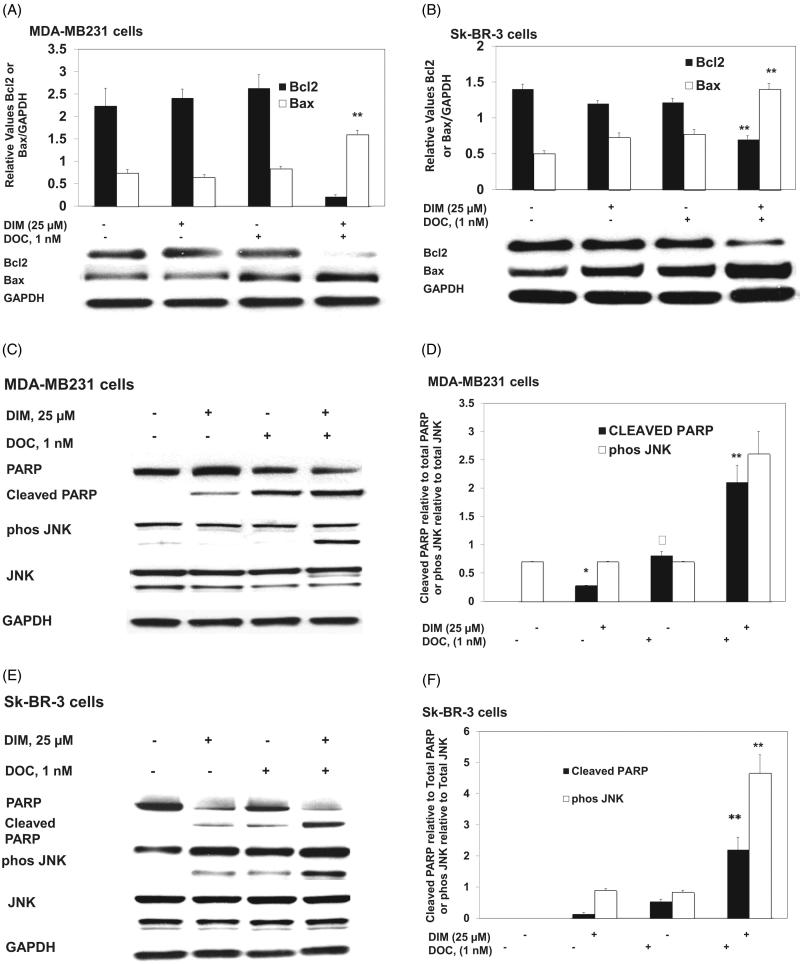
DIM in combination with DOC altered protein expression of Bcl2 and Bax, induced PARP cleavage, and activated JNK. Cells were treated with 25 µM DIM, 1 nM DOC, or 25 µM DIM plus 1 nM DOC for 48 h and probed with antibodies to Bcl2, Bax, PARP, phosphorylated JNK, and JNK. Blots were stripped and reprobed with antibody to GAPDH to verify equal loading. Bcl2 and Bax protein expression are shown in (A) MDA-MB231 cells and (B) SkBR3 cells. PARP, phosphorylated JNK, and JNK are shown in MDA-MB231 cells (C, D) and SkBR3 cells (E, F). *p* Values were determined using ANOVA. Bars represent mean scanning units ± SE of three different experiments. Bars with different symbols are significantly different (*, *p* < 0.05 vs. control; **, *p* < 0.05 vs. control, 25 µM DIM alone; **†**, *p* < 0.05 vs. control, 25 µM DIM, and DOC).

To determine whether the anti-survival effect of the DIM plus DOC combination was attributed to an increase in apoptosis, we measured cell death by annexin V analysis. MDA-MB 231 and Sk-BR-3 cells were treated with DIM, DOC and the combination of both agents for 48 h. Docetaxel alone induced a significant increase (*p* ≤ 0.05) in early apoptotic cells in MDA-MB 231 cells (12.7-fold) (Figure 1(C,D)) but not in Sk-BR-3 cells compared with control cells. Sk-BR-3 cells did not respond to DOC treatment, which like other HER2 overexpressing cells are resistant to taxanes (Yu et al. 1996; Carpenter and Lo 2013). The combination of 25 µM DIM with 1 nM DOC enhanced early apoptosis to a greater extent than DOC alone in MDA-MB231 cells (*p* ≤ 0.01) and in Sk-BR-3 cells (*p* ≤ 0.0.05) (Figure 1(E,F)). Although apoptosis was evident in both cell lines treated with the combination of 25 µM DIM with 1 nM DOC, the percent of apoptotic cells was less in the Sk-BR-3 cells (27.6%) than in the MDA-MB231 cells (45.1%). As mentioned above, this may be attributed to the increased expression of HER2 in Sk-BR-3 cells, which confers resistance to apoptosis (Fink and Chipuk 2013). Increasing the concentration of DIM from 25 to 50 µM or combining it with DOC did not further augment apoptosis in either cell line, which suggests no additional effects of combining the higher concentration of DIM (50 µM) with DOC. Western blot analyses indicated that the protein expression of Bcl-2 decreased and Bax increased in the combination group for MDA-MB231 cells (Figure 2(A)) and Sk-BR-3 cells (Figure 2(B)). Bcl-2 is an anti-apoptotic while Bax enhances apoptosis; the decrease ratio of Bcl-2:Bax promotes apoptosis. Cleavage of PARP, a characteristic of apoptosis, was also evident with the 25 µM DIM plus 1 nM DOC treatment in MDA-MB231 cells (Figure 2(C,D)) and Sk-BR-3 cells (Figure 2(E,F)) compared to control and or either agent alone.

DIM in combination with DOC activates JNK, which contributes to apoptosis. We investigated whether the stress signaling pathways were involved in mediating the synergistic cytotoxic effect of the combination treatment. JNK is activated by toxic chemical treatments, oxidative stress or environmental stress. We found that the combination of DIM with DOC increased phosphorylated JNK in comparison to DIM or DOC treatments alone with no change in JNK protein (Figure 2(C–F)). These observations suggest that activation of JNK may be one of the mechanisms mediating the synergistic cytotoxic effects of the combination. The anti-survival effect of DIM in combination with DOC is associated with increased ROS production.

**Figure 3.**
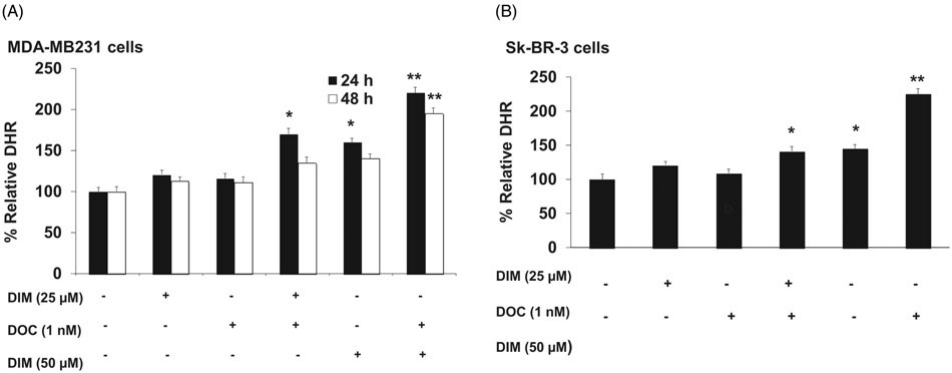
DIM in combination with DOC stimulates ROS production in DOC treated breast cancer cells. (A) MDA-MB231 and (B) SkBR3 cells were treated for 24 or 48 h with the indicated concentrations of DIM with or without 1 nM DOC and analyzed for DHR fluorescence by flow cytometer. Bars represent relative percentage of surviving cells in four independent experiments. *p* Values were determined using ANOVA. Bars with different symbols are significantly different (*, *p* < 0.05 vs. control, 25 µM DIM alone, and DOC alone; **, *p* < 0.05 vs. control, 25 µM DIM alone, DOC alone, 25 µM DIM plus DOC, and 50 µM DIM).

Next, we determined whether the increased ROS was involved in enhancing the cytotoxic effect of the combination of DIM and DOC. Previous studies have reported that DIM induced apoptosis and ROS production in breast cancer cells (Xue et al. 2005; Gong et al. 2006; Roy et al. 2008). ROS levels were evaluated by flow cytometry after staining with DCFDA, which is oxidized by ROS in the presence of endogenous peroxidase to DCF, a highly fluorescent compound. Figure 3(A,B) shows that after 24 h of treatment with 25 µM of DIM plus 1 nM DOC, ROS production increased by 46.5% in the MDA-MB231 and by 29.3% in Sk-BR-3 cells in comparison with control, 25 µM DIM alone and 1 nM DOC alone. The lower production of ROS in Sk-BR-3 cells may be attributed to the increased expression of HER2 in these cells. HER2 overexpression has been shown to decrease ROS production by preventing malondialdehyde formation and increasing SOD activity and glutathione levels (Victorio et al. 2014; Belmonte et al. 2015). Increasing the concentration of DIM to 50 µM in combination with 1 nM DOC produced a greater increase in ROS than the combination of the lower concentration of DIM with DOC. Combining the higher dose of DIM with DOC also maintained the elevation of ROS after 48 h, which suggests that the increase in ROS is dose-dependent. We also observed that the elevation in ROS levels observed after 24 h of treatment with 25 µM DIM plus 1 nM DOC in MDA-MB231 cells was not maintained at 48 h, which may be attributed to the antioxidant enzymes that remove free radicals such as copper and zinc (Cu/Zn)SOD, catalase and glutathione peroxidase. Based on our findings that the effect of the combined treatment was evident at 24 h and not at 48 h in MDA-MB231 cells, we elected to measure the effect of the treatments on ROS at 24 h in Sk-BR-3 cells.

**Figure 4.**
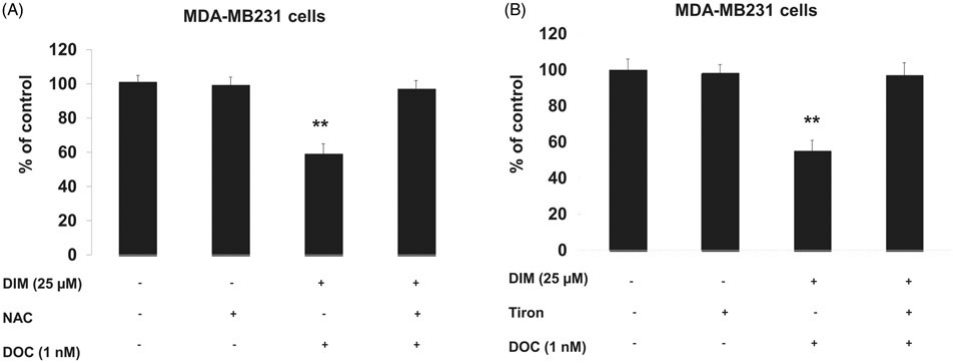
Pretreating MDA-MB231 cells NAC or Tiron abrogated the anti-survival effect of the DIM plus DOC combination. Cells were pretreated for 1 h with either (A) 5 mM NAC or (B) 1 mM Tiron and incubated for 48 h. Viability was determined by MTT assay. Bars represent relative percentage of surviving cells in four independent experiments. *p* Values were determined using ANOVA (**, *p* < 0.01 vs. control, NAC or Tiron alone, and 25 µM DIM plus DOC plus NAC).

**Figure 5.**
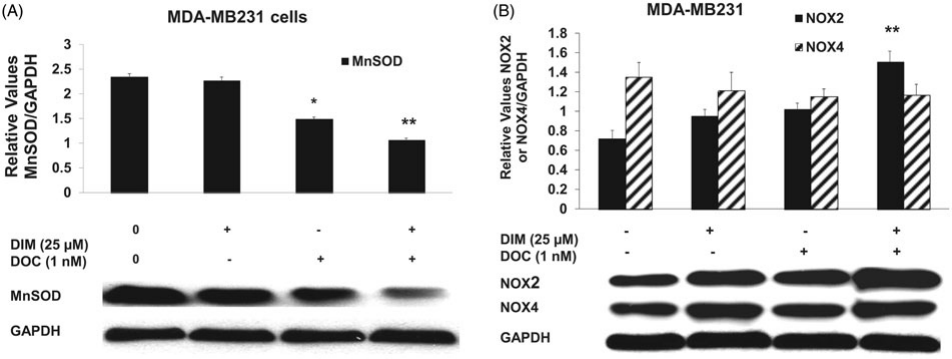
The combination of DIM and DOC decreased MnSOD and increased NOX2 protein expression. MDA-MB231 cells were treated for 48 h with 25 µM DIM with or without 1 nM DOC. Cell proteins were detected by Western blot with antibodies to (A) MnSOD, (B) NOX2, and NOX4. Blots were stripped and reprobed with antibody to GAPDH to verify equal loading. Blots were quantified by Image J and normalized to GAPDH. Bars represent mean scanning units ± SE of three different experiments. *p* Values were determined using ANOVA (*, *p* < 0.05 vs. control and 25 µM DIM alone; **, *p* < 0.01 vs. control, 25 µM DIM alone, and DOC alone).

To confirm whether the increased ROS was involved in the anti-survival and apoptotic response, we pretreated MDA-MB231 cells for 1 h with the antioxidant, NAC or the superoxide scavenger, Tiron and incubated cells for 48 h with 25 µM DIM, 1 nM DOC or their combination. Figure 4(A,B) illustrates that NAC or Tiron abolished the anti-survival effect of the drug combination, which suggests that the cytotoxic effects of the DIM plus DOC combination can be attributed to ROS. We did not conduct a similar experiment with the higher dose of DIM (50 µM) in combination with DOC. Since the pretreatment amounts of NAC or Tiron are sufficiently high to remove ROS, it is likely that the results would also indicate that ROS contributes to the anti-survival effect of the higher dose of DIM with DOC. DIM in combination with DOC decreased protein expression of MnSOD and increased NOX2. MnSOD is considered as one of the most important antioxidant enzymes in the mitochondria that can contribute to an imbalance of the redox state with increased production of superoxide (O_2_^–^) and hydrogen peroxide (H_2_O_2_)_._ Mitochondria are a major source of O_2_^–^, which increases ROS production by reacting with nitric oxide to produce peroxynitrite, a highly reactive compound (Indo et al. 2015). Superoxide is considered to be the major source of ROS in the cell. Manganese superoxide dismutase converts O_2_^–^ into H_2_O_2_, which is further removed by catalase, glutathione peroxidase and peroxiredoxins. The accumulation of H_2_O_2_ in cells promotes increased proliferation and tumor growth in cancer cells. We evaluated whether combining DIM with DOC would alter protein expression of MnSOD and NADPH oxidases. Figure 5(A) shows that DIM alone did not alter protein expression of MnSOD. DOC treatment decreased the expression by 36% (*p* < 0.05) compared to control and DIM-treated cells, whereas the combination of DIM and DOC decreased MnSOD expression by 54% (*p* < 0.01) compared to control and DIM treatment.

NADPH oxidases are also the major contributors of ROS production and regulate proliferation and cell death (Block and Gorin 2012). DIM in combination with DOC produced a significant increase (47%, *p* ≤ 0.01) in NOX2 protein expression compared with the control, DIM alone and DOC alone groups (Figure 5(B)). NOX4 protein expression was not altered, which may suggest that it is not involved in the enhanced production of ROS induced by the combination of DIM with DOC.

## Discussion

Besides its toxic effects in treating breast cancer, resistance to DOC occurs because the drug is not efficient in blocking activated survival pathways. Using nontoxic plant compounds to improve DOC efficiency and reduce toxic side effects is an attractive strategy. In this study, we present data supporting the premise that DIM improved the anti-cancer effects of DOC in breast cancer cells. Other reports found that DIM increased the effectiveness of DOC in lung cancer (Ichite et al. 2009) and paclitaxel in gastric cancer (Jin et al. 2015). The enhanced chemo-sensitivity of DIM is not limited to the taxanes. Several reports have demonstrated that DIM potentiated the effects of cisplatin in ovarian cancer (Kandala and Srivastava 2012) and gemcitabine in pancreatic cancer (Banerjee et al. 2009). Recently DIM has been shown to improve sensitivity of breast cancer cells to ionizing radiation (Wang et al. 2016), which further demonstrates the therapeutic potential of DIM in cancer treatment.

The combination of DIM plus DOC targeted ROS, Bcl2, Bax and NOX2, which were not altered by either treatment alone. Cleavage of PARP was observed in cells treated with DIM or DOC and this effect was significantly enhanced by the combination of both compounds. DIM alone and DOC alone increased protein expression of phosphorylated JNK to a similar extent but the combination of both treatments produced a much greater increase, which occurred in a synergistic manner.

In the present investigation, we observed that the combination treatment increased ROS after 24 h, resulting in apoptosis at 48 h. Since excessive production of ROS contributes to apoptosis, we evaluated whether the elevation in ROS after 24 h of treatment with DIM plus DOC led to reduced cell survival. The antioxidants NAC or Tiron abrogated the anti-survival effect of the DIM plus DOC combination, which suggests that the increased ROS observed at 24 h may trigger signaling events that promote the decreased cell survival observed with the combined treatment at 48 h.

Several studies have demonstrated that ROS mediates apoptosis through downstream activation of p38 MAPK and JNK (Benhar et al. 2002; Shen and Liu 2006; Zhu et al. 2014). We observed that the increase in ROS production with the combination treatment was associated with activation of JNK. Previous studies have demonstrated that DIM increased ROS production and induced JNK and p38 signaling in breast cancer cell lines (Xue et al. 2005; Gong et al. 2006; Roy et al. 2008). The early increase in ROS and subsequent activation of p38 or JNK may be sufficient to induce apoptosis after 48 h of treatment without sustaining the elevation in ROS. Further studies are needed to investigate the role of ROS-mediated activation of p38 and JNK in the enhanced apoptotic effect of the DIM plus DOC combination.

The increased accumulation of ROS results from the imbalance of the antioxidant scavenger enzymes such as MnSOD that remove ROS and the NOX system that produces ROS. Superoxide is the major source of ROS and, if not cleared by MnSOD, can lead to additional free radicals leading to DNA damage and cell death such as apoptosis, autophagy and necrosis (Indo et al. 2015). This is the first study to find that the DIM plus DOC combination decreases MnSOD protein expression. We found that the expression of MnSOD decreased to a greater extent with the combination treatment than with DIM or DOC alone, which was associated with increased ROS. Highly invasive MDA-MB231 cells and HER2 positive Sk-BR-3 cells have elevated levels of MnSOD compared to ER + breast cancer cell lines (Kattan et al. 2008). Suppression of MnSOD expression with antisense RNA increased H_2_O_2_ and reduced proliferation and tumor growth (Kattan et al. 2008). Their findings support the premise that suppression of MnSOD by the combination treatment is a contributory factor to the accumulation of ROS, apoptosis and decreased tumor growth. Additional studies will be important to investigate the regulatory role of MnSOD in increasing ROS with the combination treatment.

The mechanisms by which the DIM plus DOC combination regulate MnSOD are not known. A previous study has demonstrated that the transcription factors, Sp1 and NF-κB are involved in the regulation of MnSOD (Becuwe et al. 2014). DIM and DOC have been shown to increase apoptosis by inactivating NF-κΒ (Rahman et al. 2007). Since the promoter for MnSOD has binding sites for these transcription factors, the inactivation of NF-κΒ or downregulation of Sp1 could explain the decrease in MnSOD expression.

NOX2 is expressed in breast cancer cells (Satooka and Hara-Chikuma 2016). However, the role of NOX2 in mediating the effects of the chemotherapeutic drugs in breast cancer has not been explored. This is the first study to show that DIM in combination with DOC increased NOX2 expression along with elevating ROS, while the individual compounds had no effect. A recent study demonstrated that the combination of erlotinib with ampelopsin induced apoptosis and increased ROS production through the up-regulation of NOX2 (Hong et al. 2017). Further studies are needed to investigate the role of NOX2 in ROS production in breast cancer. Inhibiting NOX2 with siRNA may provide insight as to whether the increase in NOX2 contributes to the accumulation of ROS induced by the DIM plus DOC combination.

In summary, we found that DIM significantly increases the sensitivity of triple negative and HER2 positive breast cancer cell lines to DOC by decreasing cell survival and inducing apoptosis in a synergistic manner. The anti-survival and pro-apoptotic effect of combining DIM with DOC was correlated with decreased Bcl2, increased Bax, activation of JNK and cleavage of PARP. This study is also the first to show that the combination treatment induced ROS. Pretreating the cells with NAC or Tiron abrogated the cytotoxic effect of the DIM plus DOC combination, which suggests that ROS mediates the anti-survival and apoptotic effects of the treatment.

Our findings also show that the induction of ROS observed with the combination of DIM and DOC was associated with decreased protein expression of MnSOD and increased NOX2. All together, the data obtained from this study suggest a potential benefit for breast cancer patients and provide a rationale for further clinical investigation of DIM with chemotherapy for breast cancer treatment.
